# Poultry production under threat: Framework to counter misinformation and protect food security

**DOI:** 10.1016/j.psj.2025.106050

**Published:** 2025-10-31

**Authors:** Farid S. Nassar

**Affiliations:** Department of Animal and Fish Production, College of Agricultural and Food Sciences, King Faisal University, Al-Ahsa, Saudi Arabia

**Keywords:** Digital channels, Food security, Misinformation, Poultry production, Sustainability

## Abstract

Poultry industry is a fundamental pillar of global food systems, supporting nutrition, economic stability, and livelihoods worldwide. However, this sector faces significant challenges in today’s rapidly evolving media landscape, largely due to the widespread dissemination of misinformation across both traditional and digital channels. Such misinformation not only distorts public perception and influences consumer behavior but also undermines production practices, posing a direct threat to industry sustainability and food security. This study aims to explore the impact of media misinformation on the poultry industry and identify mechanisms for effectively addressing this phenomenon while enhancing food security. A qualitative descriptive approach was employed, based on a systematic review and critical analysis of previous literature, including scientific research, industry reports, and relevant publications, to identify sources of misinformation and evaluate available strategies for mitigation. Findings indicate that misinformation negatively affects the poultry sector, impacting both producers and consumers. This leads to eroded trust and influences decision-making within the industry. The results underscore the urgent need for a coordinated, multi-stakeholder approach that combines scientific rigor, proactive monitoring, and media literacy campaigns. Poultry science programs in higher education are expected to play a pivotal role, serving as hubs for professional training, the development of digital ambassadors, and the dissemination of accurate, real-time information. The study recommends implementing an integrated framework that links poultry science programs with higher education institutions, the industry sector, and governmental and non-governmental organizations to effectively detect, counter, and mitigate the impact of misinformation. By enhancing public trust, increasing consumer engagement with poultry products, improving production reliability to reduce operational and economic risks, and supporting sustainable growth by integrating these factors within a long-term framework, these measures are expected not only to safeguard the integrity of the poultry sector but also to strengthen global food security. Continuous evaluation and adaptation of these strategies remain essential to address ongoing developments in the media landscape and ensure the long-term sustainability of the industry.

## Introduction

Poultry sector and poultry meat hold significant value for both producers and consumers, offering a range of advantages. For producers, these benefits include rapid growth to market weight, relatively low input requirements, simple housing needs, and year-round production potential. Consumers, on the other hand, benefit from poultry meat’s high bioavailability, consistent availability, affordability compared to red meat, favorable nutritional profile, and appealing taste. Poultry meat serves as an excellent source of high-quality protein with low collagen content, contains beneficial unsaturated fatty acids, essential B vitamins, notably thiamine, vitamin B6, and pantothenic acid, and important minerals such as iron, zinc, and copper. Its low levels of sodium and cholesterol make it particularly suitable for health-conscious diets, aligning with modern consumer preferences for lean, nutritious meat (Petracci et al., 2014). Reflecting these advantages and the growing demand for healthier food options, global production and consumption of poultry meat have risen substantially in recent years (Popova, 2017). Since 2015, poultry has become the most widely produced and consumed meat worldwide. In 2018, global consumption of sheep and goat meat reached 15 million tons, cattle meat totaled 72.3 million tons, and poultry meat production reached 121.6 million tons. Projections for 2025 estimate further growth to 17.2 million tons for sheep and goat, 75.5 million tons for cattle, and 130.3 million tons for poultry meat (OECD/FAO, 2018).

Technology has advanced rapidly, making communication through media easier and more accessible. While reliable news disseminated through these channels raises public awareness, inaccurate information can cause confusion and misunderstanding. Many people tend to accept news circulating on social media without verifying its accuracy. With the growth of the poultry industry and the expansion of production, the poultry sector has become a frequent focus in media coverage. From time to time, both accurate and misleading reports about the sector appear in the media. For instance, concerns regarding the unhealthiness of poultry meat due to the rapid growth of broilers have attracted significant public attention, as these issues are often highlighted by the media (Haskaraca et al., 2022).

Global challenges related to feeding an increasing world population necessitate the adoption of new technologies grounded in research, education, and extension in animal sciences. While science remains the primary tool for providing solutions, food and agricultural issues, both globally and locally, are increasingly debated in public forums amid misinterpretation or distortion of scientific knowledge. Extremist voices often dominate these discussions with loud and aggressive rhetoric filled with misinformation, leaving the public confused and, at times, accepting exaggerations, distortions, and falsehoods as facts (Glenn et al., 2015).

With the expansion of digitalization and the widespread use of social media platforms, the dissemination of information has become faster and more accessible. However, this has also contributed to the widespread circulation of misleading and false information, whether unintentional or deliberate which called Disinformation, which is shared with the intent to mislead the public or influence their beliefs and behaviors. Such information continues to spread rapidly due to the lack of editorial or regulatory controls, impacting public opinion across multiple domains, including health, politics, and science, and resulting in significant social and economic consequences. The food and agriculture sector is a primary target of this phenomenon, given its critical importance to the economy and public health, the general lack of public knowledge about agricultural practices and food processing, and the emotional dimensions associated with food, including health, religion, culture, and identity. These factors make the sector particularly vulnerable to the spread of misinformation, which can take various forms, including rumors, misleading advertisements, conspiracy theories, false claims about food products, the politicization of scientific issues, and the use of agricultural information and policies as tools of influence (Woodside, 2024).

Misinformation in the U.S. food and agriculture sectors has far-reaching effects, influencing consumer choices, public health, and the economic stability of the agricultural industry. Social media platforms serve as the primary source for the rapid dissemination of such information, as users and content creators can easily share it without oversight or correction, while traditional media sometimes amplify or politicize it, further exacerbating the problem. People believe and spread misinformation due to being overwhelmed by large volumes of information, confirmation bias reinforced by algorithms, and the repetition of messages, which creates a sense of familiarity with the content. Human tendencies to take mental shortcuts and strong emotional reactions to novel information also contribute to its spread. Additionally, general lack of knowledge, insufficient evidence to counter rumors, and public perceptions of official sources, such as the U.S. government and traditional media, as untrustworthy, further facilitate the circulation of false information. These factors highlight the urgent need to explore solutions and strategies to mitigate the impact of misinformation on the food and agriculture sectors, protecting consumers and safeguarding the industry’s integrity (Woodside, 2024).

Strategic communication serves as a cornerstone for enabling higher education institutions to strengthen their academic reputation and enhance the quality of education. By developing effective communication practices with both internal and external stakeholders, universities can reinforce their credibility and elevate their academic standing. Moreover, strategic communication is a vital tool for managing reputation efficiently, fostering sustainable relationships, and ensuring that institutions maintain a strong and influential academic position over the long term (Nassar et al., 2024).

This study aims to examine how media misinformation affects the poultry industry and to identify practical strategies to address it while supporting food security. The study addresses three main questions:

Q1- What is media disinformation, and how does it spread and impact credibility in the context of the poultry industry?
Q2- What are the impacts of misinformation on consumer trust, economic aspects, and the sustainability of the poultry industry?
Q3- What strategies can be used to counter misinformation and protect the poultry industry’s reputation and sustainability?

In light of the increasing threats facing global poultry production, particularly due to the spread of misinformation through media channels, it has become essential to develop effective mechanisms to counter this phenomenon and safeguard food security. This study aims to analyze the impact of media misinformation on the poultry industry, in addition to identifying mechanisms to address it, enhance production sustainability, and improve the quality of production practices, with a direct focus on protecting food security.

Despite growing recognition of the critical role of accurate information and organized strategies in supporting the poultry sector, a clear gap remains in the research literature regarding the mechanisms currently employed to combat misinformation, as well as the lack of proactive measures capable of addressing this issue. Moreover, the integration of these mechanisms within the academic and strategic frameworks of educational programs, and the evaluation of their impact on improving student experience, enhancing production competencies, and empowering the next generation of poultry industry professionals, remains largely underexplored.

Given the frequent difficulty in discerning the intent behind information, and in order to simplify this complex phenomenon within the context of the poultry industry, the current study adopts the term “misinformation” comprehensively to refer to any information that ultimately proves to be false. This approach allows for a thorough discussion of the mechanisms and impacts associated with the spread of false information on social media and other communication contexts. It also enables the identification of effective strategies and mechanisms to counter misinformation, enhance production sustainability, and improve the quality of production practices, while maintaining a direct focus on protecting food security.

Furthermore, misinformation negatively affects decision-makers, consumers, and producers alike, highlighting the urgent need for coordinated efforts among academic, research, and non-academic institutions to develop comprehensive mechanisms aligned with modern digital advancements. The study recommends implementing a structured framework that integrates these mechanisms to ensure the dissemination of accurate information, strengthen the integrity and resilience of poultry production, build public trust, and contribute directly to protecting food security. Additionally, the study emphasizes the importance of practically evaluating the implementation of these mechanisms in real-world industry settings and continuously monitoring developments in the media landscape to ensure the effectiveness and sustainability of the adopted measures.

## Material and methods

This study employs a qualitative descriptive research methodology to systematically explore the impact of media misinformation on the poultry industry and to identify mechanisms that can effectively counter misinformation while enhancing food security. Qualitative descriptive research, as described by Neuman (2014), is particularly suited for this study as it allows for “presenting a picture of the specific details of a situation, social setting, or relationship” and “begins with a well-defined issue or question and endeavors to describe it accurately,” with a focus on “how” and “who” inquiries. Lambert and Lambert (2012) further emphasize that qualitative research designs, including phenomenology and grounded theory, can serve both descriptive and explanatory purposes, and they argue that the designation "qualitative descriptive research" accurately characterizes the research approach without mislabeling it with methodologies such as phenomenology, grounded theory, or ethnography. In addition, qualitative descriptive research “often derives from naturalistic inquiry, which asserts a dedication to examining phenomena in their inherent state as much as feasible within the research context” (Lambert and Lambert, 2012).

Given these considerations, this inherently qualitative study not only explores the impact of media misinformation on the poultry industry but also systematically identifies mechanisms to counter misinformation while enhancing food security, thereby establishing both methodological rigor and originality in the approach. To achieve this, a comprehensive content analysis of both electronic and printed materials related to the events under investigation was conducted, as recommended by Bowen (2009). Content analysis involves three critical phases, skimming, thorough reading, and interpretation, which enable the systematic categorization of textual data into smaller, meaningful, and manageable units (Weber, 1990) and facilitate the identification of recurring themes and patterns to uncover underlying meanings (Patton, 2002). This ensures analytical depth beyond mere description.

The methodology followed a structured, four-phase approach to strengthen rigor and enhance analytical synthesis. Phase one involved a systematic search across leading academic databases, including Google Scholar, Scopus, and Web of Science, using targeted keywords such as “Misinformation,” “Fake News,” “Media,” “Poultry Production,” and “Higher Education.” The search focused on peer-reviewed articles, institutional reports, and empirical studies published in English over the past five years, supplemented by foundational literature to ensure comprehensive scholarly support. Additionally, phase two established clear inclusion and exclusion criteria, with studies included if they addressed the definitions and characteristics of misinformation and its impact on the poultry industry and food security, while studies lacking empirical evidence, methodological rigor, or direct relevance to higher education or production environments were excluded to ensure analytical clarity.

Moreover, Phase three involved systematic data extraction and qualitative synthesis, focusing on each study’s objectives, key findings, identified threats arising from misinformation, and the effectiveness of proposed counter strategies. This phase went beyond descriptive summarization, employing thematic synthesis to integrate findings across sources, identify patterns, differences, sectoral vulnerabilities, and critical points of intervention, thereby demonstrating analytical depth and originality. Phase four consisted of an in-depth examination of the synthesized findings to explore how misinformation can be addressed through integrated frameworks that combine evidence-based scientific communication, technological innovation, and continuous collaboration among all relevant stakeholders, including poultry science programs in higher education institutions, government agencies, commercial poultry production companies, professional and cooperative farmer associations, and consumers. This phase extends the analysis from description to strategic application, proposing actionable frameworks designed to strengthen sector resilience, protect food security, and maintain consumer trust in the face of evolving misinformation threats.

By integrating descriptive, interpretative, and strategic elements within this structured, four-phase methodology, the study not only provides a rigorous and comprehensive assessment of how misinformation threatens poultry production but also ensures analytical synthesis, methodological depth, and originality. This framework addresses previous limitations, offering evidence-based strategies for mitigating risks and safeguarding food security while maintaining the credibility and relevance of the research.

## Results

### The rise and complexity of misinformation in the digital era

The sharp rise in the circulation of fake news is considered one of the most pressing challenges of the current cultural era (Liffreing, 2016). In particular, the influence of social media platforms, particularly their capacity to disseminate information in real time, has significantly exacerbated this problem (Broda and Strömbäck, 2024). Furthermore, modern digital technologies have enabled the widespread production, distribution, and consumption of fake news and misinformation, making it increasingly difficult to distinguish between accurate and misleading information (Kalsnes, 2018). Notably, fake news is often shared on social media more frequently than articles published in traditional editorial media, which typically apply some form of gatekeeping and verification (Adjin-Tettey, 2022). Moreover, Caplan et al. (2018) confirmed that platforms such as Facebook and Twitter have played a significant role in facilitating the spread of fake news, highlighting both its rapid dissemination and the potential harm it may cause.

To better understand this phenomenon, it is important to consider the conceptual distinctions among related terms. Specifically, there is a range of terms and concepts associated with misinformation, primarily differing based on several factors, including the author’s intent to mislead the audience and the factual basis of the content. In general, Most of these terms can be categorized into three major types of information disorder (Wardle and Derakhshan, 2017): misinformation, which refers to false information spread without the intent to mislead, such as unintentionally sharing a story containing outdated information; disinformation, which refers to false information deliberately created to cause harm, such as publishing news with manipulated statistics; and malinformation, which refers to truthful information disseminated with the intent to cause harm, such as circulating a statement out of its original context to mislead the audience. Additionally, other related concepts frequently discussed in the literature include rumors, which are stories or statements of unverified factuality that may turn out to be partially true or entirely false (Zubiaga et al., 2016), and fake news, which refers to fabricated information intended to deceive by mimicking the form of legitimate news (Lazer et al., 2018).

In this context, fake news is defined as a news article that are intentionally and verifiably false, and could mislead readers (Broda and Strömbäck, 2024). It is also noteworthy that, it is sometimes referred to as information pollution (Wardle and Derakshan, 2017), media manipulation (Warwick and Lewis, 2017), or information warfare (Khaldarova and Pantti, 2016). Furthermore, in UNESCO’s handbook for journalism education and training, authored by Ireton and Posetti, disinformation is defined as deliberate (often orchestrated) attempts to confuse or manipulate people by delivering false information, whereas misinformation refers to misleading information created or disseminated without manipulative or malicious intent (Ireton and Posetti, 2018). In addition, there is also malinformation, which involves the intentional dissemination of truthful information usually by changing context, date, or time for personal or corporate purposes rather than public interest (Broda and Strömbäck, 2024).

Regarding the differences, the two main differences between misinformation and disinformation are: 1) fake news mimics the form of mainstream news (Zimdars and McLeod, 2020), whereas misinformation does not; and 2) while disinformation is purposefully crafted to mislead the audience, those spreading misinformation do not do so intentionally, as they are unaware that the information they share is fabricated or false. Overall, fake news, disinformation, and malinformation collectively produce misinformation. Importantly, disinformation and malinformation are particularly dangerous because they are deliberately orchestrated and resourced by malicious actors and reinforced by digital technologies and platforms, including social networks (Ireton and Posetti, 2018).

Although these phenomena are not new, fake news, misinformation, and disinformation are not new phenomena, they have received significant attention in contemporary times, and fake news has been classified as one of the major threats to society. Specially, fake news and disinformation take various forms, including satirical news websites, fabricated news items, manipulated images, propaganda, false press releases (Tandoc et al., 2018), as well as sensationalist tabloid content (Marwick, 2018). Moreover, technological advancements that have facilitated the spread of misinformation have also increased its complexity and made it harder to control. Tools such as bots, deep fake videos, and other forms of digital manipulation have rendered misinformation more sophisticated and difficult to detect. Additionally, bots can amplify false content by rapidly sharing and liking it, creating the illusion of widespread consensus (Shao et al., 2018). A prominent example is the emergence of highly realistic Deepfakes videos generated by artificial intelligence, which have significantly complicated efforts to detect and counter false information. Consequently, these advanced technologies make it increasingly difficult for traditional verification methods to keep pace with such ongoing developments (Chesney and Citron, 2019).

Furthermore, the spread of misinformation across social media networks is considered a major factor that generates confusion within communities and can sometimes lead to ineffective responses during crises and disasters (Zhai et al., 2024). In particular, these platforms also serve as fertile ground for the circulation of unverified messages, thereby accelerating the dissemination of misinformation (Procter et al., 2013). According to Uncertainty Reduction Theory, individuals seek information as a means of alleviating ambiguity and forming a clearer understanding of unfolding events. Moreover, literature indicates that such behavior intensifies as levels of uncertainty increase (Neuberger and Silk, 2016), and experimental findings show that individuals facing threats that heighten uncertainty are more inclined to seek additional information related to those threats (Goodall and Reed, 2013).

On the other hand, on social media and digital platforms, misinformation or fake news is disseminated at an extremely rapid pace, making it difficult to retract or control. Even more concerning is that the identity of the person spreading such information is often unknown or hard to trace, unlike traditional print or electronic media where authors are identifiable and accountable for any harm caused. In addition, with the rapid proliferation of misinformation and harmful content at the click of a button, technology companies face significant challenges in regulating such content on social media while simultaneously balancing public responsibilities, definitions of free speech, and identifying both content and source. In this context, content creators on social media often remain anonymous, as the content typically lacks identifying information, and even if the creator is eventually traced, potentially after the content has been shared millions of times, the damage may already have occurred (Ohwadua, 2022). As a result, audiences are frequently exposed to inaccurate content, whether they are aware of it or not, including fake news and misinformation, which can lead consumers to feel confused and uncertain about the accuracy of their own knowledge (Rapp and Salovich, 2018). For example, s study found that fake news left the majority of Americans 64 % confused about basic facts (Barthel et al., 2016).

### The impact of misinformation on consumer behavior, poultry production, and market stability

It is evident that misinformation has a significant impact on both consumers and the poultry sector. A substantial proportion of consumers reported being influenced by false information circulating in the media, negatively affecting their purchasing decisions and the overall perception of the industry. This highlights a clear gap in consumer awareness and understanding of proper poultry production and rearing practices, emphasizing the urgent need to provide accurate, scientifically backed information to correct misconceptions. Moreover, there is a critical need to raise awareness among poultry producers regarding best rearing practices and to improve existing systems to reduce the number of uninformed consumers and producers, thereby mitigating the negative effects of misinformation (Haskaraca et al., 2022). Accordingly, these findings reflect the profound impact of misinformation on the behaviors of both consumers and producers in the poultry sector. The harm is not limited to individual decision-making but extends to affect the overall image of the industry and its production efficiency. This underscores that enhancing communication mechanisms and providing accurate, reliable scientific information is not merely an option but a strategic necessity to raise awareness among all stakeholders, ensure the sector’s sustainability, and safeguard the supply chain from the negative repercussions of false information.

On the other hand, the poultry sector in India constitutes a vital component of the country’s GDP, growing at an annual rate of 8–10 %. Its value reached USD 22.97 billion in 2022, with projections estimating it to reach USD 41.94 billion at a compound annual growth rate of 10.18 % between 2023 and 2028. The broiler poultry sector was negatively affected by misinformation on social media, leading to declines in both prices and consumption. For instance, during 2019–2020, rumors and false claims circulated regarding broiler chicken, including assertions that its consumption could transmit the coronavirus, cause early puberty in girls, or lead to ovarian cysts and menstrual disorders in women, alongside reports of virus infection in chickens in Bangalore. Swift interventions by health authorities within two days of these claims, coordinated with the government and producers through media coverage, helped mitigate the impact of misinformation and ensured that consumers continued purchasing broiler chicken despite the circulating rumors (Kathiravan, 2024).

In addition, during the global COVID-19 pandemic, India witnessed a widespread surge of rumors and misinformation falsely linking the consumption of chicken and eggs to the transmission of COVID-19 to humans. This misinformation fueled public fear, resulting in a sharp decline in poultry consumption, followed by a significant drop in demand and prices. Furthermore, with the implementation of nationwide lockdowns, the poultry industry’s crisis deepened due to disruptions in the supply of feed and essential health inputs, forcing producers to cull large quantities of eggs, chicks, and birds. This led to severe economic losses estimated at approximately USD 3,053 million, highlighting the direct negative impact of misinformation on one of India’s most critical food sectors (Kolluri et al., 2021).

As a result, although the poultry sector constitutes a vital pillar of economic output in countries like India, misinformation can create significant disruptions within this industry. Social media plays a clearly influential role in rapidly amplifying false information, making it difficult to control or correct without urgent intervention from relevant authorities. Specifically, misinformation regarding the health risks of broiler chicken, such as claims of coronavirus transmission or other health hazards, has fueled consumer fear, leading to a sharp decline in demand and prices. This sudden downturn has not only affected product consumption but has also disrupted the entire supply chain, halting the delivery of feed and essential health inputs and forcing producers to cull large quantities of eggs, chicks, and birds, resulting in substantial economic losses. Consequently, this situation highlights the direct negative impact of misinformation on market stability and sector productivity, as it undermines trust between consumers and producers, increases economic risks for farmers, and weakens the industry’s capacity to respond to emergency crises. Moreover, the rapid spread of such information through digital platforms presents an additional challenge in controlling misleading content, making the poultry sector more vulnerable to fluctuations in demand and prices and threatening its long-term economic sustainability.

In the United States of America, the annual number of foodborne illness cases is estimated at approximately 47.8 million, with raw poultry being the most frequently associated source. Despite the risk of cross-contamination, washing raw poultry remains a common practice among consumers. For example, a study involving 1,822 participants revealed that 73.5 % reported washing raw poultry, and 68.1 % were unaware that this practice is unsafe. After being exposed to the U.S. Department of Agriculture’s awareness message, 81.9 % of previously uninformed consumers expressed moderate to high confidence in their ability to stop washing poultry. However, some consumers continued the practice despite knowing the correct behavior, believing that simply cleaning surfaces afterward was sufficient to prevent contamination. These findings highlight a significant gap in consumer awareness, emphasizing the need for more effective educational campaigns to correct misconceptions, influence behavior, and promote food safety (Vatral et al., 2022).

Additionally, a study by Sonntag et al. (2019) revealed that consumers in the European Union exhibit low acceptance of modern, intensive poultry production systems, primarily due to growing concerns over animal welfare. The study also showed that consumers’ actual knowledge of modern poultry production varied widely, ranging from detailed specialized understanding to misinformation. This misinformation played a key role in shaping negative attitudes toward intensive production systems, as consumers relied on unscientific interpretations suggesting that modern poultry farming involves extensive use of antibiotics, negatively affecting meat quality and posing health risks. Furthermore, consumers were largely unaware of improvements in animal welfare, such as the EU-wide ban on conventional cages for laying hens, which amplified the impact of knowledge gaps and misinformation in reducing acceptance of these modern systems. Moreover, perceived problems in the poultry value chain were largely attributed to the industrial farming system, while farmers were mostly exempted from blame (Sonntag et al., 2019).

Similarly, Estes et al. (2015) indicated that consumers in Arkansas exhibit a high degree of uncertainty regarding the safety of conventionally produced poultry, particularly concerning the presence of antibiotics and hormones, reflecting the ambiguity of the information available to them about traditional production practices. Their perception of most poultry farms as large-scale industrial operations further heightens this concern and influences their purchasing behavior. In contrast, organic poultry is perceived as healthier, reflecting consumers’ tendency to seek alternatives considered safer. Moreover, Laconi et al. (2023) highlighted a clear disparity in the implementation of biosecurity measures between farmers and advisors on Italian poultry farms. While farmers perceive the implementation as relatively high, primarily focusing on their own farms, advisors, who possess a broader perspective encompassing multiple farms and production categories, consider the implementation to be less effective.

On the other hand, the circulation of misinformation in developing countries has a significantly greater impact compared to its effects in developed nations. Poultry farming plays a crucial role in improving livelihoods in rural areas by providing better nutrition and additional income. Accurate information is essential for enhancing poultry management and welfare in these communities; however, access to such information is limited due to a shortage of agricultural extension personnel, low awareness levels, weak infrastructure, and frequent power outages. Farmers rely heavily on informal sources, such as family, neighbors, local extension services, and traditional media, primarily seeking information that directly affects poultry health and welfare. There is an urgent need to strengthen agricultural extension services, raise farmers’ awareness of available information sources, and facilitate reliable access to information to ensure that farmers receive accurate and trustworthy guidance. This, in turn, can improve poultry productivity and support sustainable livelihoods in rural communities (Msoffe and Ngulube, 2016).

## Discussion

### The impact of misinformation on trust and practices in the poultry industry

Since fake news incidents can "harm and hurt the reputation" of a brand and may escalate rapidly into full-blown crises, therefore, communication professionals have adopted a range of strategies to mitigate their effects (Jahng et al., 2020). However, given the considerable reputational risks, handling fake-news crises extends beyond routine operational practices and requires a strategic communication approach (Jahng et al., 2020). Specifically, according to the latest definitions, strategic communication "encompasses all communication that is substantial for the survival and sustained success of an entity" and more precisely, it refers to "the purposeful use of communication by an organization or other entity to engage in conversations of strategic significance to its goals" (Zerfass et al., 2018).

Moreover, the spread of rumors can be driven by a complex interplay of emotions and factual content, while efforts to correct such rumors often rely on objective facts. However, the transmission of emotions between individuals during the circulation of rumors can significantly reduce the effectiveness of these corrections, as intense feelings tend to reinforce the acceptance and entrenchment of misinformation among the public (Zeng and Zhu, 2019). Consequently, emotional appeals serve as a powerful tool for information dissemination, facilitating faster and more widespread social transmission.

In the context of the poultry industry, studies have shown that negative social media posts regarding the Newcastle Disease outbreak in Southern California between 2018 and 2020 attracted a wider audience and generated greater engagement compared to positive posts, highlighting the profound impact of negative sentiment on farmers’ and the general public’s awareness of poultry-related health risks (Gendreau et al., 2022). This phenomenon demonstrates that while negative content can increase attention and engagement, it also carries a significant risk of amplifying fear and spreading misinformation if not managed and addressed strategically and effectively. Furthermore, authority cues, such as whether the source is a government official or an expert, significantly influence individuals’ perceptions of message credibility, making the identity of the sender a crucial factor when messages are retransmitted on social media. Typically, domain experts, celebrities, and institutional actors receive more mentions and reports compared to ordinary users, particularly on social media platforms (Zhang et al., 2014).

Similarly, the number of followers on a social platform also serves as an important differentiator among organizational sources, celebrities, and ordinary users, and is considered an indicator of a user’s popularity or influence, reflecting the size of their audience. Similarly, Chua et al. (2017) found that both the number of rumor retweets and rumor-correction retweets are positively associated with the number of Twitter followers. In social media environments, follower status is also regarded as a marker of credibility; for instance, Margolin et al. (2018) found that fact-checking messages carry greater weight when posted by someone a user follows. Importantly, the credibility of information is the most critical factor in enhancing farmers’ practices in poultry farming, as it directly influences their decisions and production behaviors. Limited or moderate exposure among farmers may lead to incorrect practices, highlighting the need to strengthen the reliability of communication channels, expand extension programs, and address the shortage of field personnel. Ensuring that farmers have access to accurate and scientifically verified information is essential for improving production efficiency and sustaining the poultry sector (Bhuyian et al., 2013).

Moreover, the poultry industry faces significant challenges due to the widespread circulation of fake news and misinformation, which misleads both producers and consumers. For producers, false information regarding breeding methods, nutrition, vaccination, or disease control can lead to unscientific practices, increasing the risks of reduced productivity, disease outbreaks among poultry, and financial losses. At the consumer level, rumors and false information about the safety of poultry products or production methods erode market trust, potentially decreasing demand and raising doubts about product quality.

In terms of poultry science programs, misinformation hinders students and experts from distinguishing between facts and falsehoods, leading to misguided educational and advisory decisions and undermining the credibility of available scientific data. Moreover, the rapid dissemination of misleading information through social media and the difficulty of tracing its sources exacerbate these problems, making it challenging to control or correct false content before damage occurs. Consequently, these challenges are multidimensional, directly impacting production quality, consumer trust, and the efficiency of training and advisory programs in the poultry sector.

In the context of the poultry sector, credibility emerges as a decisive factor in shaping farmers’ decisions and production behaviors, directly influencing their practices. Limited or moderate exposure to information among farmers may lead to inappropriate practices, underscoring the need to strengthen the reliability of communication channels, expand extension programs, and address the shortage of field personnel. Therefore, ensuring that farmers have access to accurate, scientifically

### The multidimensional threat of misinformation on poultry industry sustainability and food safety

It becomes evident that misinformation has a substantial negative impact on both the poultry industry and food safety. The high number of annual foodborne illness cases, with raw poultry as the most common source, underscores the sector’s vulnerability to improper consumer behaviors. The continued widespread practice of washing raw poultry, despite well-known cross-contamination risks, indicates the prevalence of misconceptions and a lack of consumer awareness. Thus, this demonstrates that misinformation affects not only knowledge but also practical consumer behavior, increasing the likelihood of health risks associated with poultry products. Overall, this phenomenon highlights the significant gap in consumer awareness and illustrates the ongoing negative effects of misinformation on public trust in poultry products and the overall performance of the industry.

In light of this, it is evident that misinformation profoundly affects the poultry industry by shaping negative consumer attitudes toward modern, intensive production systems, reducing their acceptance, and heightening concerns over product safety and animal welfare. This distorted awareness leads to fluctuations in demand and prices, undermines the sector’s competitiveness, increases economic pressures on producers, and constrains investment and innovation. Moreover, Misinformation also shifts blame toward the industrial system rather than the farmers and erodes trust in regulatory and scientific institutions, complicating crisis management and the restoration of supply chain stability. Overall, misinformation represents a multidimensional threat to the poultry industry: it does not merely alter perceptions but reshapes demand, influences regulatory policies, weakens innovation and investments, exposes the supply chain to simultaneous economic and health risks, and erodes the institutional trust necessary for effective risk management and crisis recovery.

In this context, it becomes evident that incomplete or misleading information has a compounded impact on the effectiveness of biosecurity measures in the poultry industry. The gap between farmers’ and advisors’ perceptions reflects a genuine knowledge deficit. Consequently, such differences in perception can lead to misjudgment of risks, where farmers may believe they are sufficiently protected while sector-level gaps leave the industry vulnerable to disease outbreaks and infection transmission. Furthermore, insufficient awareness based on accurate information exacerbates the gap between local practices and comprehensive scientific recommendations, limiting the effectiveness of preventive strategies and increasing both the economic and health risks for the sector.

As a result, the spread of misinformation in developing countries, particularly in rural areas, can have a profound effect on the poultry industry. Rural farmers often depend on informal sources for guidance on poultry health and welfare. This reliance on unreliable sources fosters misconceptions about production practices, undermines the implementation of biosecurity measures, and diminishes farmers’ health awareness. Additionally, the lack of extension services and weak infrastructure exacerbates this knowledge gap, limiting farmers’ ability to make informed decisions, reducing productivity, and compromising poultry welfare. Thus, the negative impact of misinformation on the poultry industry is reflected in increased health and economic risks and the erosion of sustainable livelihoods in rural communities.

In the poultry industry, it is evident that misinformation represents a widespread and multidimensional threat at both national and international levels. Its impact extends beyond shaping consumer perceptions, directly influencing purchasing behaviors, producer practices, supply chain stability, and overall sector productivity. Specifically, false or incomplete information, whether related to health risks, production methods, animal welfare, or biosecurity measures, creates a state of uncertainty among both consumers and producers, undermining trust, distorting demand, and destabilizing markets.

Moreover, in developed countries, such misinformation can skew public perceptions of conventional and intensive production systems, reduce their acceptance and limiting investment in modern practices. In contrast, in developing countries, however, it exacerbates knowledge gaps and restricts access to reliable guidance, leaving farmers more vulnerable to economic and health risks. Additionally, the rapid spread of misinformation through digital and informal channels further complicates these challenges, making swift intervention and effective communication essential. Accordingly, addressing this phenomenon is not merely optional but a strategic imperative. If left unchecked, misinformation threatens not only economic outcomes but also public trust, food safety, and the sector’s capacity to respond to crises. This underscores the urgent need to implement coordinated, evidence-based strategies to mitigate its effects and safeguard the sustainability of the poultry industry.

### Strategies to counter misinformation and protect the poultry industry’s reputation and sustainability

The rapid spread of misleading and false information in recent years has raised increasing concerns within academic and industrial circles, as this phenomenon has become one of the most complex and pressing issues. This is primarily due to the accelerated pace of technological development, which has made manipulating information and disseminating it widely through social media platforms both easy and low-cost. The matter is further complicated by factors linked to human psychology and sociology, making it difficult to understand users’ motives, predict their behavior, or prevent them from engaging in the dissemination of such information. Thus, combating disinformation is not a short-term task but rather a long-term challenge that requires persistent efforts. Individual scientific disciplines alone cannot provide sufficient solutions; instead, a multidisciplinary approach is essential. Within this context, a continuous race unfolds between malicious actors and defenders: while modern technologies may provide advanced tools to detect or limit the spread of disinformation, they can also be exploited by malicious users to accelerate its dissemination. Since the tools themselves are neutral, the outcomes ultimately depend on who uses them. This ongoing battle is expected to last for a long time, making the pursuit of continuous and rigorous efforts critically important (Barni et al., 2022).

The impact of misinformation is not limited to the academic or scientific field alone; it also poses a significant threat to corporate communication strategies. Today, organizations are not only tasked with delivering their messages and strengthening their image but are also compelled to defend their reputation against false narratives that may cause them serious harm (DiFonzo and Bordia, 2007). The situation becomes even more critical when disinformation campaigns target major corporations, political groups, or even individuals, often leading to public relations crises that demand substantial resources to manage. Given the high potential for rapid and widespread dissemination of misinformation across digital platforms, organizations must remain vigilant and proactive by adopting advanced strategies for early detection and effective response to such challenges (Tucker et al., 2018).

In the poultry sector, effective communication among all stakeholders emerges as a fundamental step in addressing the negative effects of misinformation on production at both local and international levels. For instance, Cadena et al. (2018) reported that the collaboration network among backyard poultry trainers in California suffers from weak internal connectivity, which reduces the efficiency of cooperation among different stakeholders. In developing countries, combating misinformation among consumers and producers faces a set of intertwined challenges, most notably the lack of sufficient resources and capacities to achieve effective communication, in addition to the relatively low use of mass communication or media channels. Furthermore, farmers’ personal characteristics, such as educational level, farm size, and participation in training programs, play a decisive role, as many poultry farmers in rural areas tend to rely primarily on individual communication channels as their main source of agricultural information relevant to poultry production. This underscores the urgent need to enhance awareness and training programs and to design communication strategies tailored to the needs of these farmers, thereby promoting the adoption of agricultural innovations and improving the efficiency of information dissemination within the poultry farming community (Hossain et al., 2021).

In this context, it becomes evident that misinformation represents a major strategic challenge capable of inflicting direct harm on both consumers and producers in the poultry industry. Therefore, developing effective strategies to detect, counter, and limit its spread is of paramount importance, particularly in light of the enormous volume of data and the rapid pace at which it circulates across digital platforms, which makes real-time monitoring and effective response increasingly difficult. Addressing such massive volumes of information requires advanced technological tools and adequate human and material resources to ensure efficient management.

Adding to this complexity is the weak coordination among stakeholders in the poultry industry, which constitutes a fundamental challenge in responding to rumors and misinformation. Effective confrontation of this issue requires close and integrated collaboration among poultry science programs, commercial companies, government agencies, technology firms, and consumers. However, conflicting interests and diverging priorities among these actors often result in fragmented efforts with limited impact, further complicating the response process. Hence, the success of poultry science programs in combating misinformation depends on joint and well-coordinated actions among all relevant stakeholders to formulate a unified strategy capable of safeguarding the sustainability of the industry, strengthening its competitiveness, and building trust between the sector and consumers. This includes the following:

***Enhancing media literacy and professional training to counter misinformation in the poultry industry****.* Integrating media and information literacy components into the curricula of poultry science programs represents a strategic step to keep pace with the rapid developments in digital media and social media platforms. These programs play a central role in empowering students and professionals to develop critical thinking skills and accurately evaluate information, thereby enhancing their ability to confront false news and rumors that could harm the poultry sector.

Poultry science programs bear the responsibility of continuously updating their curricula to align with technological and media advancements, while also providing advanced practical and professional training opportunities in agricultural extension and poultry management. This training particularly targets youth and university students who require broader awareness of the career opportunities available in this vital sector. Additionally, these programs contribute to building specialized capacities that enable practitioners and researchers to monitor and analyze rumors and respond to misinformation campaigns through training workshops and field simulations of real-world scenarios. They also develop reliable scientific content that is directed directly to consumers via digital platforms. Through this integrated role, poultry science programs become a cornerstone in preparing scientific and professional personnel capable of critically analyzing information, enhancing public trust in poultry products, ensuring the sustainability of the sector, and supporting its credibility in the long term.

***Educational Campaigns to Enhance Media Literacy among Consumers and Poultry Farmers*.** Poultry science programs are not merely academic curricula taught at universities; they represent a strategic pillar capable of protecting the poultry production sector from the risks of misinformation and rumors. These programs have the unique ability to combine scientific rigor with practical awareness, making them well-positioned to lead extensive efforts to raise awareness among both farmers and consumers. Within this framework, poultry science programs play a central role in leading educational campaigns aimed at enhancing media literacy among consumers and farmers, thereby improving their ability to critically evaluate information and reducing their exposure to misleading news that could harm the sector. These campaigns seek to empower individuals to develop cognitive skills that enable them to question false news and slow its spread, positively contributing to industry protection and fostering public trust. This includes designing and implementing specialized training programs for students, professionals, and sector workers, as well as raising public awareness through workshops, educational courses, and informational materials that emphasize critical thinking and fact-checking skills.

Recognizing the cultural and educational diversity among communities, poultry science programs adapt these campaigns to suit target audiences, particularly in developing communities or among individuals with limited formal education, by creating simplified tools and easily understandable presentation methods that address local challenges. These programs also design media and information literacy initiatives that provide effective outreach to individuals outside formal education. By collaborating with specialists in media, communication, and information technology, poultry science programs ensure that educational and awareness messages reach audiences effectively, enhancing the literacy of consumers and farmers and enabling them to distinguish between accurate and misleading information, thereby reinforcing the programs’ essential role in safeguarding the sustainability and credibility of the poultry production sector.

***Proactive Monitoring and Forecasting to Counter Misinformation.*** Poultry science programs, in collaboration with poultry production institutions, contribute to establishing specialized units to monitor and track news and misinformation related to the poultry sector in real-time, targeting both consumers and farmers. This collaboration relies on integrating scientific data available at universities with market information provided by companies, aiming to identify potential risks before they reach the public. Proactive monitoring and intelligent forecasting are essential tools that enable these institutions to effectively combat misinformation by observing the media environment, understanding how events and messages are presented on media outlets and social media platforms, and analyzing how the audience perceives these messages.

The collaboration between universities and production institutions includes implementing real-time monitoring mechanisms, promoting transparency and open communication, and maintaining continuous coordination with all stakeholders to enhance institutional resilience and limit the spread of misinformation. This partnership also relies on artificial intelligence and machine learning algorithms to analyze digital data and predict rumors before they spread widely, enabling institutions to launch proactive corrective campaigns that reduce economic damage while preserving reputation and public trust. These tools allow for tracking misinformation patterns and the speed of its dissemination on digital platforms, providing precise insights into potential threats and supporting the implementation of effective countermeasures, thereby strengthening the role of collaboration between poultry science programs and production institutions in protecting and sustaining the poultry sector.

***Digital Ambassadors for Real-Time Counteraction of Rumors and Misinformation.*** Digital ambassadors act as an interactive bridge connecting consumers, the poultry production sector, and poultry science programs. These academic programs facilitate the creation of an integrated network of students, experts, and trained farmers who serve as digital ambassadors responsible for delivering accurate scientific information about the poultry industry through social media platforms. Poultry science programs train these digital ambassadors to use engaging and interactive methods, such as short videos, infographics, and podcasts, to convey scientific facts grounded in research and practical experience, making them easier to understand and raising consumer awareness of the sector’s vital role in food security and sustainable nutrition. Furthermore, each ambassador is directly linked to proactive monitoring and forecasting systems to counter misinformation, enabling them to be the first to receive alerts about rumors and respond swiftly with reliable, real-time content. This approach helps limit the spread of misinformation and builds trust between the public, poultry science programs, and the production sector.

In addition, poultry science programs can develop a digital platform that allows the public to submit inquiries directly about agricultural products and poultry production processes. Artificial intelligence can be used to provide immediate answers to frequently asked questions, while more complex inquiries are referred to experts at universities and research centers to ensure accuracy and reliability. Through this integrated approach, poultry science programs enhance transparency, strengthen trust between consumers and the poultry industry, and highlight the central role of academic programs in disseminating scientific knowledge and effectively countering digital misinformation.

## Conclusion

Poultry sector is a fundamental component of global food security; however, it faces increasing challenges due to the rapid spread of misinformation and disinformation through traditional and digital media. The results of this study indicate that misinformation negatively affects producers, consumers, and educational institutions by undermining trust, influencing decision-making, and compromising production practices, resulting in tangible economic losses, reduced consumer confidence, and complicating efforts to achieve sustainable sector growth, thereby highlighting the urgent need for effective and proactive countermeasures. The study further indicates that combating misinformation requires a multi-dimensional and proactive approach that combines scientific rigor, technological innovation, and continuous coordination with all stakeholders.

Moreover, specific recommendations include empowering poultry science programs in higher education to serve as hubs for professional training, media literacy campaigns, and the development of digital ambassadors capable of disseminating accurate, real-time information, enhancing proactive monitoring and predictive analysis capabilities while implementing targeted communication strategies to counter misinformation before it spreads widely; and establishing an integrated framework linking academia, industry, government, and technology to strengthen sector resilience, protect its reputation, and maintain consumer confidence.

The current study also highlights the novelty of the findings by presenting an integrated model that combines academic, technological, and social dimensions to proactively address misinformation, and demonstrates the importance of these measures for stakeholders, including policymakers, educational institutions, producers, and consumers, by providing them with the tools necessary to make evidence-based decisions and guide practices to ensure sector sustainability. Furthermore, continuous evaluation and adaptation of strategies remain essential in a rapidly evolving media environment, enabling the poultry sector to respond effectively to emerging challenges, maintain sustainable growth, and ensure the availability of safe, nutritious, and reliable poultry products, thereby directly contributing to the enhancement of global food security.

## Funding

This work was supported by the Deanship of Scientific Research, Vice Presidency for Graduate Studies and Scientific Research, King Faisal University, Saudi Arabia (Grant No. KFU253351).

## CRediT authorship contribution statement

**Farid S. Nassar:** Writing – review & editing, Writing – original draft, Visualization, Validation, Supervision, Software, Resources, Project administration, Methodology, Investigation, Funding acquisition, Formal analysis, Data curation, Conceptualization.

## Disclosures

The author declares that there are no conflicts of interest regarding the publication of this paper.
